# Clinical and experimental treatment of advanced melanoma with a focus on immunotherapy

**DOI:** 10.1093/cei/uxaf073

**Published:** 2025-12-01

**Authors:** Lucy Millar, Rafael Di Marco Barros, Matthaios Kapiris, Christos Nikolaou, Marco Gerlinger, Yin Wu

**Affiliations:** Centre for Inflammation Biology and Cancer Immunology, King’s College London, London, UK; Barts Cancer Institute, Queen Mary University of London, London, UK; Centre for Inflammation Biology and Cancer Immunology, King’s College London, London, UK; Department of Oncology, Guy’s Hospital, London, UK; Centre for Inflammation Biology and Cancer Immunology, King’s College London, London, UK; Department of Oncology, Guy’s Hospital, London, UK; Comprehensive Cancer Centre, King’s College London, London, UK; 4th Oncology Clinic, Henry Dunant Hospital, Athens, Greece; Barts Cancer Institute, Queen Mary University of London, London, UK; Centre for Inflammation Biology and Cancer Immunology, King’s College London, London, UK; Department of Oncology, Guy’s Hospital, London, UK

**Keywords:** Melanoma, Cancer Immunology, Immunotherapy, Immune Checkpoint Inhibitors

## Abstract

Melanoma is currently the fifth most common cancer in the UK, and its incidence is rising. Although surgery is curative for many early-stage tumours, advanced disease which is inoperable has historically also been considered incurable. Recent and rapid advances in cancer immunotherapy, particularly immune checkpoint inhibitors, have now revolutionized the management of advanced melanoma with many patients likely being cured. Here, we review the immunobiology of melanoma, the growing list of standard-of-care immunotherapies and the considerations around which treatment regimen to use. We also review evidence from recent clinical trials of promising novel immunotherapies which will hopefully help patients who do not benefit from current treatments.

## Teaser

Immunotherapy has revolutionised outcomes for patients with advanced melanoma. Here we review the immunobiology of melanoma, current standard-of-care treatments and evidence for experimental immunotherapies.

## Introduction

Melanomas are malignant neoplasms arising from neural crest-derived, pigment-producing cells called melanocytes. These cells are predominantly found in the lowermost layer of the epidermis and in hair follicles where they protect keratinocytes from DNA damage by producing the ultraviolet radiation (UV)-absorbing pigment, melanin [1]. Outside of the skin, melanocytes can also be found within the anogenital and sinonasal mucosa and the uveal tract. Thus, melanomas are categorized into cutaneous, mucosal, and uveal subtypes based on the primary tissue of origin [1]. This review will focus on cutaneous melanomas which account for over 90% of all cases.

Whilst early-stage cutaneous melanomas can be cured by surgery, patients with advanced and inoperable melanomas have historically had extremely poor prognoses with median overall survival (mOS) of just 6–9 months and 5-year overall survival of ∼5% [2]. Until recently, the standard-of-care (SoC) treatment for patients with inoperable disease was chemotherapy with dacarbazine or dacarbazine-containing regimens. Response rates were modest (∼10–30%) and short-lived (∼3–4 months), and multiple phase 3 trials of combination therapies over the preceding four decades failed to demonstrate significant improvement in overall survival [2]. Set against this dismal backdrop, current therapies have revolutionized the management of cutaneous melanomas with 5-year survival rates for patients diagnosed with inoperable disease today approaching 50% [3–5]. Whilst targeted therapies directed against activating BRAF mutations (found in ∼40% of cutaneous melanomas) have played a role [6–10], it is modern immunotherapies which have delivered the bulk of long-term survival gains [11–13]. Here we review immunosurveillance in melanoma, current SoC immunotherapies, the limitations of these treatments and promising therapies on the horizon.

### Melanoma and immunosurveillance

**Figure 1:**
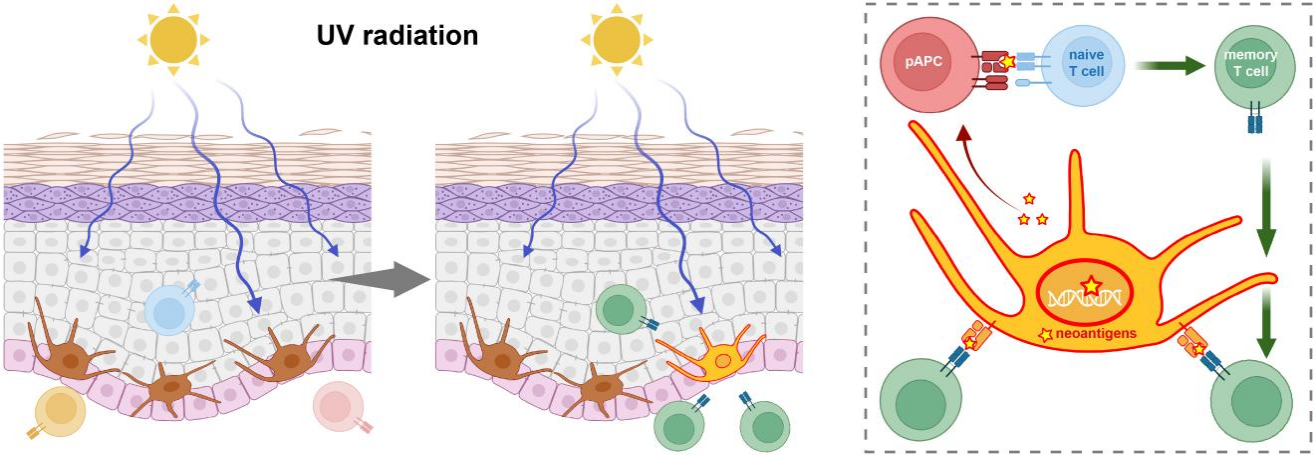
Melanoma and T cell immunosurveillance. Ultraviolet radiation from chronic sun exposure induces genomic mutations in long-lived melanocytes found in the basal layer of the epidermis. Non-synonymous mutations may generate ‘neoantigens’ that can be loaded onto self-MHC. As neoantigens are somatically acquired, they de facto escape central tolerance and are likely to also evade peripheral tolerance. Thus, accumulated neoantigens in melanoma cells increase the cells visibility to antigen-specific αβ T cells. Created in BioRender. Wu, Y. (2025) https://BioRender.com/bbudcuu.

Despite the lacklustre efficacy of combination chemotherapy regimens, many oncologists remained optimistic about the potential benefit of immunotherapy for melanoma as multiple lines of evidence suggested a protective role for the immune system. UV radiation is the greatest environmental risk factor for the development of cutaneous melanomas and is estimated to account for over 90% of cutaneous melanomas [14]. UV radiation also results in the accumulation of genomic mutations, predominantly cytosine-to-thymine substitutions [15]. Thus, cutaneous melanomas have one of the highest tumour mutational burdens of all cancers [15]. These somatic mutations, which include both oncogenic driver mutations and passenger mutations, can serve as neoantigens for engagement of tumour-reactive T cells (Fig. 1). Also consistent with a role for immunosurveillance, patients who are immunosuppressed (e.g. solid organ transplant recipients) have significantly higher rates of melanoma than expected, which is not the case for some other common cancers (e.g. breast cancer, prostate cancer) [16]. Moreover, approximately 10–30% of surgically resected early-stage melanomas are associated with a phenomenon termed regression [17–19]. Histologically, regression is characterized by reduced viable melanoma cells accompanied by fibrosis and an inflammatory immune infiltrate [19]. Thus, regression is thought to reflect spontaneous and effective immunological rejection of melanoma. Intriguingly, patients with evidence of melanoma regression may be more likely to respond favourably to subsequent immunotherapy [20, 21].

### Immunoediting and immune escape

**Figure 2:**
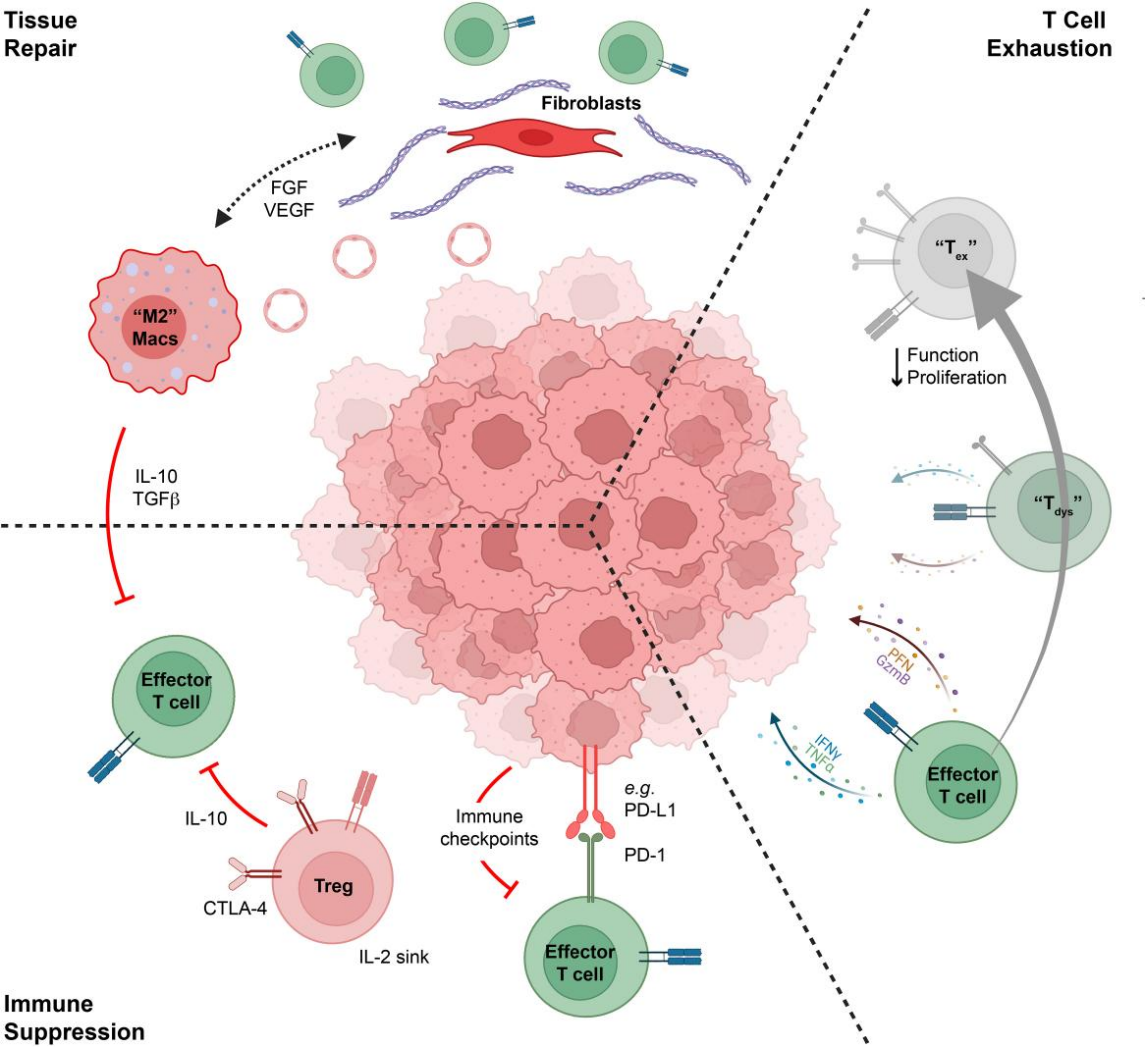
Chronic inflammation within the TME engages physiological tolerance mechanisms. Most human cancers develop over many years. During this time, chronic inflammation within the tumour microenvironment (TME) engages tissue-intrinsic physiological tolerance mechanisms that have evolved to prevent autoimmunity. Alternatively activated ‘M2’ macrophages orchestrate concurrent attempts at wound healing by recruiting fibroblasts and endothelial cells. In the context of cancer, fibroblasts and endothelial cells can favour the growth and persistence of tumours by excluding cancer-rejecting effector T cells and supporting neovascularization. M2 macrophages also secrete anti-inflammatory cytokines (e.g. IL-10, TGFβ, etc.) which suppress effector T cell function. Chronic inflammation also recruits regulatory T cells (Tregs) which can suppress effector T cells through secretion of immunosuppresive cytokines, sequestration of co-stimulation by CTLA-4 and/or consumption of IL-2. Chronic activation of effector T cells has been shown to cause progressive dysfunction and eventually terminal exhaustion in these cells. This is characterized by an initial, reversible impairment in cytokine production/cytolytic capacity as well as proliferation in ‘early-dysfunctional’ (‘Tdys’) T cells followed by an irreversible state of terminal exhaustion (‘Tex’) in which the cells are no longer functional even in the presence of therapeutic immune checkpoint inhibitors. Created in BioRender. Wu, Y. (2025) https://BioRender.com/3upc37h.

Tumour neoantigens arise from somatic mutations and therefore bypass central and (to a lesser extent) peripheral immunological tolerance. Thus, the presence of neoantigens should improve the immunological visibility of tumours to the adaptive immune system [22]. Whilst this likely underpins cases of spontaneous regression seen in early-stage melanomas, advanced tumours have clearly evaded physiological immunosurveillance. Indeed, clinically evident tumours have not only evolved under immune selection, but these tumours have also shaped the immune response in a process proposed by Schreiber and colleagues known as immunoediting [23]. In this model, most nascent cancers are eliminated by immunosurveillance before they are clinically detectable. However, some tumours will progress to establish an equilibrium with immunosurveillance by one of two main routes. Firstly, the genetic instability of cancers drives the development and outgrowth of subclones with myriad immunoevasive capabilities [24, 25]. Secondly, and in part aided by cancer-intrinsic immunoevasion, chronic inflammation within the tumour microenvironment engages multiple physiological tissue tolerance mechanisms [24] including active immune suppression, progressive T cell dysfunction/exhaustion and concurrent attempts at tissue repair (Fig. 2). Together, these processes initially permit tumour persistence (equilibrium) and subsequently allow for growth and immune escape as the balance shifts away from cancer elimination. Cancer immunotherapies aim to shift this balance back in favour of immune clearance [26].

### Immune exhaustion and immune checkpoint inhibitors

Chronic antigen-specific stimulation of T cells, particularly αβ T cells, has been shown to drive these cells down a stepwise path towards a state of exhaustion [27, 28]. This phenotype is characterized by the upregulation of inhibitory immune checkpoint receptors and a progressive impairment in proliferative capacity and effector function (Fig. 2). In melanoma, this exhaustion phenotype is enriched in tumour-reactive CD4+ and CD8+ αβ T cells which demonstrate increased expression of checkpoints including PD-1, CTLA-4, TIGIT, LAG3, TIM3, and CD39 [29–31]. Whilst true terminal exhaustion in T cells is difficult to reverse, the preceding ‘early-dysfunctional’ state (aka ‘progenitor’, ‘stem-like’) has proven to be more amenable to therapeutic reprogramming through blockade of inhibitory immune checkpoints by monoclonal antibodies [32–34]. These immune checkpoint inhibitors (CPIs), particularly ones targeting PD-1, CTLA4 and LAG3, have revolutionized the treatment of many cancers and have been especially effective in the treatment of melanoma.

## Current treatment of advanced melanoma

Surgery remains the gold standard treatment for early-stage melanomas with cure rates of 95% or more for patients with thin tumours (<4 mm or <2 mm with ulceration) and no evidence of locoregional spread [35]. For patients with thicker tumours and/or those with locoregional spread (in-transit and/or lymph node metastases), relapse rates after surgery with curative intent can range anywhere from ∼20% to ∼70% [36–38].

After several decades as standard-of-care, chemotherapy is now rarely used in the management of advanced melanoma. Patients with tumours harbouring activating BRAF V600 mutations (∼40% of cutaneous melanomas) now have the option of treatment with targeted MAP-kinase (BRAF±MEK) inhibitors. Response rates to combination BRAF/MEK inhibitors are very high (∼60–70%) with responses occurring as soon as a few days after initiation of treatment [7, 39, 40]. Thus, these drugs have proven particularly useful when rapid control of disease is required, for example in cases where vital organs are threatened or where there is large volume disease. Although important in the management of BRAF-mutant melanomas and reviewed elsewhere [41], BRAF/MEK inhibitors are rarely curative in the advanced disease setting. On the other hand, CPIs have the potential for cure and have emerged as the clear gold-standard first-line treatment for advanced melanomas regardless of BRAF mutational status [11–13].

### CPI therapies

#### Anti-CTLA-4

Ipilimumab, a fully human monoclonal IgG1 antibody targeting the cytotoxic T-lymphocyte associated protein 4 (CTLA-4) immune checkpoint, was the first CPI licensed for the treatment of advanced melanomas. Although initially trialled in the second-line(+) setting [42] where it had relatively modest response rates of ∼10–15%, ipilimumab soon became the SoC first-line therapy owing to its impressive durability and potential for cure in most patients who responded. A pooled analysis of survival data from patients with advanced melanoma treated with ipilimumab found ∼20% of patients were long-term survivors with a clear watershed at the 3-year mark [43]. Moreover, compared with earlier immunotherapies such as high-dose IL-2, ipilimumab was far more tolerable [44]. Ipilimumab is thought to work by two main mechanisms. The first and more widely accepted mechanism is via inhibiting the sequestration of CD80/CD86 (B7.1/B7.2) by CTLA-4 and thus restoring co-stimulation via CD28 to T cells [45–47]. This may be particularly important for the critical naïve-to-memory licensing checkpoint of tumour-antigen reactive T cells in addition to more broadly increasing activation of memory T cells through restoring co-stimulation. Indeed, patients treated with ipilimumab have a broader peripheral T cell repertoire after therapy, consistent with increased licensing of naïve T cells [48, 49]. A second and intriguing putative mechanism relates to the IgG1 isotype of ipilimumab. IgG1 can engage low affinity Fc receptors on NK cells and other immune cells to activate antibody-directed cellular cytotoxicity against target cells [50]. Despite its name, CTLA-4 is expressed at much higher density on regulatory T cells (Tregs) compared with conventional effector and cytotoxic T cells. An elegant study in mice found that treatment with an IgG1 anti-CTLA-4 ipilimumab analogue preferentially depleted intratumoural Tregs in an Fc receptor dependent manner and that Treg depletion was linked to effective tumour control [51]. Consistent with this, patients with melanoma who also had Fc receptor polymorphisms associated with higher IgG1 affinity were more likely to survive after ipilimumab therapy [51].

#### Anti-PD-1

Hot on the heels of ipilimumab, two pivotal phase III studies demonstrated the superiority of monoclonal antibodies blocking the PD-1 immune checkpoint (pembrolizumab and nivolumab) in head-to-head trials versus ipilimumab [52, 53]. KEYNOTE-006 randomized patients with inoperable melanoma to receive either pembrolizumab or ipilimumab [52] whilst CheckMate 067 randomized patients to nivolumab alone, combination ipilimumab/nivolumab or ipilimumab alone [53]. Both KEYNOTE-006 and CheckMate 067 found that anti-PD-1 treatments were not only more effective (∼30–40% objective response rates for anti-PD-1 versus ∼15% for ipilimumab) but also more tolerable. About 15% of patients receiving anti-PD-1 experienced treatment-related moderate (grade 3) to severe (grade 3+) side effects whilst this figure was ∼20–30% for patients receiving ipilimumab. Beyond melanoma, anti-PD-1 therapies (including those targeting its ligand PD-L1) have also emerged as the CPI of choice for multiple cancer types [54–57], consistent with the central role PD-1 plays in maintaining tissue tolerance in normal physiology [58].

PD-1 is upregulated on the surface of T cells upon activation by T cell receptor engagement with cognate peptide-MHC (e.g. from pathogen-derived antigens, cancer neoantigens, etc.). In parallel, pro-inflammatory cytokines produced by activated T cells, particularly IFNγ, are potent inducers of its ligand PD-L1 on both tumour cells and tumour associated stromal cells (e.g. macrophages), as well as on normal epithelial cells [59–62]. Binding of PD-L1 to PD-1 on T cells leads to the recruitment of SHP2 protein tyrosine phosphatases to the T cell receptor (TCR) where they antagonise TCR and CD28 signalling by dephosphorylating and deactivating downstream ZAP70 [63]. Thus, the PD-1 axis is a physiological fail-safe checkpoint to restrain prolonged T cell activation within tissues. Unsurprisingly, tumours co-opt this intrinsic and ready-made tolerance mechanism from the tissues in which they arise.

#### Anti-CTLA-4, anti-PD-1, or combination?

The distinct mechanisms of action of anti-CTLA-4 and anti-PD-1 CPIs raised several important questions including which treatment is more durable and whether combination treatment could unlock synergistic gains. Given the role of CTLA-4 in the critical naïve->memory tolerance checkpoint versus the more downstream role of PD-1 in predominantly regulating licensed memory T cells responses [62], many hypothesized that anti-CTLA-4 treatment would generate more durable responses. Indeed, evidence from some murine models support this [64] but clinical trial results suggest that anti-PD-1 responses may be just as durable if not more so [3, 65].

Combination therapies in oncology have generally failed to demonstrate synergistic gains in efficacy and many combinations do not even achieve additive gains [66–68]. In other words, the response to two drugs is at best the sum of the response to each drug alone and oft times even less than this. Concurrent anti-CTLA-4/PD-1 for the treatment of advanced melanoma does not appear to have broken this trend. Whilst combination nivolumab and ipilimumab was shown to have a modest numerical advantage in terms survival compared to single agent nivolumab in CheckMate 067, the study was not powered to detect statistically significant differences between these two arms [53]. Instead, it demonstrated a significant improvement in survival of both nivolumab containing arms (single and combination) versus ipilimumab alone [3, 53, 69]. Notably, a post-hoc descriptive analysis found no difference in overall survival between patients on combination therapy and those on anti-PD-1 monotherapy [65].

Whilst no randomized phase 3 studies have demonstrated superiority of combination anti-CTLA-4/PD-1 over anti-PD-1 alone for patients with advanced melanoma, many oncologists continue to regard this combination as more efficacious. In support of this, a retrospective pooled analysis of six CheckMate clinical trials (CheckMate 003, 004, 066, 067, 069, and 511) reported a ∼10% gain in overall survival in patients receiving first-line ipilimumab/nivolumab versus those receiving nivolumab alone [70]. This study also suggests that patients with melanomas harbouring BRAF mutations and those negative for PD-L1 may be more likely to benefit from combination treatment. However, it is worth noting that retrospective pooled analyses may be confounded by differences in study designs and patient populations. Indeed, patients receiving nivolumab monotherapy in CheckMate 003 and 066 had numerically worse outcomes than the comparable cohort in CheckMate 67. Conversely, those who received ipilimumab/nivolumab combination therapy in CheckMate 004 and 511 had numerically better outcomes than the comparable cohort in CheckMate 67, potentially distorting survival in favour of combination therapy. Notwithstanding the aforementioned caveats, real-world data from two independent studies from Denmark [71] and the Netherlands [72] both suggest no overall benefit of anti-CTLA-4/PD-1 over anti-PD-1 alone. Consistent with this, randomized phase 3 evidence demonstrates no benefit in adding anti-CTLA-4 to anti-PD-1 therapy in the adjuvant setting (CPIs given after surgery to prevent melanoma relapse) [73]. Moreover, a recent meta-analysis of advanced cancers other than melanoma demonstrated no improvement in overall survival for combination anti-CTLA-4/PD-1 versus anti-PD-1 alone [74]. Thus, both single agent anti-PD-1 and combination anti-CTLA-4/PD-1 continue to be recommended as first-line therapy in advanced melanoma [75].

#### Anti-PD-1 and anti-LAG3 combination

More recently, the combination of anti-PD-1 and anti-LAG3 has been licensed as an alternative first-line immunotherapy for patients with advanced melanoma based on the results of the RELATIVITY-047 trial [76]. In this phase 2/3 trial, patients were randomized to receive either nivolumab alone or in combination with relatlimab (anti-LAG3) with patients receiving the latter demonstrating significantly improved progression-free survival (median progression-free survival of 10.1 versus 4.6 months). However, there was no significant improvement in overall survival (hazard ratio of 0.8 in favour of dual therapy but not statistically significant) suggesting that patients may be salvaged with second-line therapies at progression [77].

### Choice of first-line CPI therapy—special considerations and toxicity

Anti-PD-1 alone or in combination with either anti-LAG3 or anti-CTLA-4, is licensed and recommended as first-line treatment for patients with advanced melanoma [75]. With no formal evidence of overall survival benefit demonstrated by either combination regimen over monotherapy in unselected patient populations [69, 77], the choice of treatment requires careful tailoring to each patient [75]. Based on subgroup analyses, patients with PD-L1 negative tumours and those with more aggressive disease (e.g. raised LDH, BRAF mutations) may be more likely to benefit from the addition of either anti-LAG3 or anti-CTLA-4 to an anti-PD-1 backbone [69, 77]. Efficacy wise, a recent retrospective comparative study found little difference between anti-LAG3 and anti-CTLA-4 (in combination with anti-PD-1) with adjusted response rates of 48% and 50%, respectively [78]. For patients with brain metastases, evidence suggests that combination anti-CTLA-4/PD-1 may be superior. In a multicentre, phase 2 study of patients with asymptomatic brain metastases, anti-CTLA-4/PD-1 treatment was associated with an intracranial objective response rate of 57%, similar to that observed in extracranial lesions [79]. A smaller phase 2 study found that patients receiving anti-PD-1 monotherapy for asymptomatic brain metastases had an intracranial objective response rate of just 26%, also concordant with extracranial responses [80]. Notably, most patients in the former study were naïve to immunotherapy whilst the majority in the latter were not, and therefore likely to have been immunotherapy resistant to begin with. A more recent phase 2 study randomized patients with asymptomatic brain metastases to either anti-CTLA-4/PD-1 combination therapy (*n* = 36) or anti-PD-1 monotherapy (*n* = 27). In this study, combination therapy yielded numerically superior objective responses (51% versus 20%) and 7-year intracranial progression-free survival (42% versus 15%). On the other hand, a single arm phase 2 study which treated patients (*n* = 37) with anti-PD-1 and anti-VEGF therapy (bevacizumab) reported an objective intracranial response rate of 54.1% and an impressive 4-year overall survival of 51.6% [81].

In addition to considering individual patients’ cancer intrinsic factors (e.g. metastatic sites, PD-L1/BRAF status, LDH, etc.), the choice of anti-PD-1 monotherapy versus combination therapy requires nuanced consideration of each regimen’s toxicity profile and clinical accessibility vis-à-vis funding and/or licensing restrictions in different territories. For example, in the United Kingdom, only one line of anti-PD-1 therapy (either as monotherapy or in combination with anti-LAG3 or anti-CTLA4) is reimbursed. Thus, careful selection of patients based on subgroup analyses can help to identify those most likely to benefit from monotherapy versus combination therapy. Indeed, in Europe, the European Medicines Agency (EMA) has granted market authorization for anti-PD-1 + anti-LAG3 therapy only for patients with PD-L1 negative (<1%) tumours, reflecting the importance afforded to robust subgroup analyses.

In addition to efficacy and availability, the toxicity profile of each regimen should be a key factor in deciding between treatments. The majority of CPI treatment-related adverse events are autoimmune in nature and are referred to as immune-related adverse events (irAEs). Every organ system may be affected although the skin, gastrointestinal tract and endocrine organs are the most susceptible to irAEs [52, 53, 76].

Consistent with the role played by CTLA-4 in maintaining peripheral tolerance, (i.e. by restraining naïve-to-memory licensing), anti-CTLA-4 CPIs such as ipilimumab are generally associated with higher rates of irAEs. For example, in randomized studies of ipilimumab versus pembrolizumab or nivolumab, skin irAEs were ∼50% higher in patients treated with ipilimumab. This was even more pronounced for gastrointestinal irAEs, including diarrhoea and colitis, where ipilimumab treatment was associated with ∼3–5× increased incidence. Likewise, endocrine irAEs, such as hypophysitis, were also higher in patients treated with ipilimumab (∼5×) although thyroid dysfunction bucked this trend and was higher in patients treated with anti-PD-1 (∼2–5×) [52, 53].

Whilst combination therapy with anti-PD-1 and anti-CTLA-4 has not demonstrated synergistic efficacy in advanced melanoma, there has been some evidence to suggest synergistic toxicity in this regimen. Overall, monotherapies with anti-PD-1 inhibitors have been associated with a ∼15% rate of grade 3/3+ (moderate to severe) treatment-related adverse events versus ∼20–30% for anti-CTLA-4 inhibitors [52, 53]. Notably, CheckMate 067 in which patients were randomized 1:1:1 to receive either nivolumab, ipilimumab, or ipilimumab/nivolumab, reported grade 3/3+ treatment-related toxicities in 16.3%, 27.3%, and 55% of patients, respectively. Perhaps a more tangible metric was the rate of discontinuation of therapy due to treatment-related toxicities at 7.7% for nivolumab, 14.8% for ipilimumab, and 36.4% for the combination [53].

Combination anti-PD-1/LAG-3 therapy has recently emerged as a viable alternative to anti-CTLA-4/PD-1 for patients keen to achieve the best possible chances of remission. In addition to significant improvements in progression-free survival, combination anti-PD-1/LAG-3 also had numerically superior objective response rates compared to anti-PD-1 alone (43.1% versus 32.6%). Importantly, the addition of anti-LAG-3 was associated with only a modest increase in grade 3/3+ treatment-related toxicity versus anti-PD-1 monotherapy (18.9% versus 9.7%) [76].

### TVEC oncolytic viral therapy

Until recently, the only other licensed immunotherapy for the treatment of advanced melanoma was the oncolytic virus, talimogene laherparepvec, or TVEC. TVEC is a modified herpes simplex virus rationally engineered to preferentially kill cancer cells whilst simultaneously engaging an adaptive immune response. Deletion of the ICP34.5 gene in TVEC removes its ability to antagonize host anti-viral responses mediated by RNA-regulated protein kinase (PKR) and thus confers selectivity for tumour cells which often lack PKR activity. In addition, deletion of the ICP47 genes hampers the ability of TVEC to inhibit antigen presentation whilst addition of a GM-CSF transgene payload increases local accumulation of antigen presenting dendritic cells [82]. Unlike CPIs, TVEC is directly injected into accessible skin or nodal deposits of melanoma. Response rates to TVEC in the first-line setting (∼30%) approach those of anti-PD-1 monotherapy, albeit with benefit mostly in patients without visceral metastases (e.g. stage IIIB-IVM1a) [83]. Consistent with its potential to engage systemic immune responses, the benefits of TVEC can be both durable and seen in distant, untreated lesions [83–85]. Moreover, as TVEC is delivered locally, it is very well tolerated with few grade 3/3+ systemic toxicities [83]. Thus, it is an excellent option for patients for whom the risk of severe autoimmune irAEs from CPIs are deemed too great. Notably, whilst the registrational study of TVEC was conducted in the first-line setting before the widespread availability of CPIs, it is licensed for use in the second-line (+) setting as well. Indeed, in patients with prior exposure to CPIs, TVEC appears to retain efficacy [86–89]. Despite this favourable toxicity to benefit profile, the logistical requirements of direct intratumoural delivery combined with a two-weekly treatment schedule have limited the adoption of TVEC to a small number of specialist centres.

### Tumour-infiltrating lymphocytes

Until 2024, palliative BRAF/MEK-targeted therapy (for BRAF V600 mutant melanomas) and/or chemotherapy were the only approved treatments available for patients with CPI-refractory disease not suitable for TVEC (e.g. metastatic disease outside of the skin and lymph nodes). In 2024, the US FDA granted accelerated approval for the first tumour-infiltrating lymphocyte (TIL) therapy (lifileucel) for patients with unresectable or metastatic melanoma previously treated with a PD-1 blocking antibody, and if BRAF V600 mutation positive, a BRAF±MEK inhibitor. Lifileucel is an autologous, polyclonal T cell product expanded from surgically resected tumours based on the pioneering work of Steven Rosenberg and colleagues [90, 91]. The phase 2 C-144–01 study treated 153 patients who had progressive melanoma despite prior anti-PD-1 therapy using lifileucel and demonstrated an objective response rate of 31% in this difficult to treat population. Notably, these responses were durable with about half of responders remaining in remission at 3 years after treatment [92]. In the UK, lifileucel is still awaiting regulatory approval with decisions expected in late 2025 or early 2026 whilst in Europe the application for marketing authorization to the EMA was withdrawn in July 2025.

A separate phase 3 study of TILs (TIL-NKI/CCIT) led by the Netherlands Cancer Institute and the Danish National Centre for Cancer Immune Therapy compared TIL-NKI/CCIT treatment versus ipilimumab in 168 patients with advanced melanoma (randomized 1:1). Notably, the majority of patients (86%) in this study were also refractory to anti-PD-1 treatment. Objective response rates favoured TIL-NKI/CCIT treatment (49% versus 21%) and progression-free survival was significantly in favour of TIL-NKI/CCIT with a hazard ratio of 0.5 [93]. Whilst not formally licensed, TIL-NKI/CCIT therapy is available in the Netherlands and Denmark via early access programmes.

Given the benefit of TIL therapy in patients with anti-PD-1 refractory melanoma, a phase III clinical trial is currently underway evaluating the efficacy of TIL + anti-PD-1 versus anti-PD-1 alone in the first-line setting (TILVANCE301, NCT05727904). This question of when to sequence TILs in melanoma treatment is particularly relevant in light of the considerable direct financial costs of TILs, indirect costs of healthcare resources (e.g. operating theatre time, intensive care unit time, etc.) and the significant toxicities associated with treatment. Indeed, nearly all patients will have a grade 3/3+ cytopenia as a result of conditioning chemotherapy. Moreover, the majority of patients will experience symptoms of an acute systemic inflammatory response (e.g. fever, hypotension, hypoxia, tachycardia, confusion, etc) collectively known as cytokine release syndrome (CRS). Up to ∼10% of patients may experience grade 3/3+ CRS which may require vasopressors and/or high-flow oxygen (≥40%), often in a high-dependency unit. Thus, the selection of patients for TIL therapy demands careful consideration of those most likely to benefit [94, 95] and is particularly pertinent in the first-line setting where a substantial proportion of patients will be cured by anti-PD-1 monotherapy alone.

## Therapies on the horizon

Although melanoma is only the fifth most common cancer in the UK and 17th globally [96], it has historically led the way for trials of novel immunotherapies. This role at the vanguard of immuno-oncology continues and below we review several promising novel immunotherapies on the horizon.

### Cancer vaccines

Given the proven efficacy of T cell centric therapies (e.g. CPIs), there has been renewed interest in therapeutic vaccination to raise T cell responses against cancer associated/specific antigens. A recent phase 1 study of an off-the-shelf mRNA vaccine targeting four unmutated melanoma-associated antigens ± anti-PD-1 demonstrated immunogenicity and efficacy in patients with advanced melanoma, nearly all of whom had prior exposure to anti-PD-1 [97]. Objective tumour regression was observable across all doses of vaccine monotherapy but most frequently occurred at the highest dose in combination with anti-PD-1 (5/10 patients). Notably, objective responses were observed even amongst patients who had previously progressed on anti-PD-1 therapy, suggesting that this approach can salvage CPI-responsiveness in refractory patients. Recent updates from a phase 2 study of a DNA vaccine encoding the melanocyte differentiation antigens TRP-2 and gp100 given in conjunction with anti-CTLA-4/PD-1 CPI therapy in the first-line setting have also been encouraging [98]. Of 43 patients treated so far, 72% had an objective response which is numerically higher than the ∼50% objective response expected from an anti-CTLA-4/PD-1 CPI backbone [78].

Rather than targeting cancer associated/specific antigens, an alternative strategy is to vaccinate against tumour promoting host factors. This latter approach is theoretically more robust to genomic instability, cancer evolution, and antigenic escape. Indeed, a well-conducted phase 1/2 trial of a peptide vaccine against indoleamine 2,3-dioxygenase (IDO) and PD-L1 given in conjunction with anti-PD-1 reported an objective response rate of 80% in 30 patients with metastatic disease [99]. Notably, many patients had poor prognostic factors at baseline including raised LDH (37%), liver metastases (33%), and more than one metastatic site (80%). Remarkably, the observed objective response rate was twice that of a similar historical cohort receiving anti-PD-1 monotherapy alone (∼40%). A confirmatory phase 3 trial has recently finished recruiting and the full results are eagerly awaited (NCT05155254).

Collectively, the results from these vaccine studies have been encouraging but nonetheless require confirmation in larger randomized studies.

### Adoptive cell therapies

Adoptive cell therapies, particularly chimeric antigen receptor T (CART) cell therapies, have demonstrated clear efficacy in treating haematological malignancies [100–104]. Apart from TIL therapy (see above), adoptive cell therapies have seen only limited success in treating solid cancers.

Unlike haematological malignancies, most solid cancers do not express enough tumour-associated/specific antigens (e.g. CD19 on B cell leukaemia/lymphomas) on their surface to permit efficient targeting by immunoglobulin-based CART constructs. One approach to circumvent this has been to make use of TCRs recognizing internal cancer-associated/specific antigens presented in the context of surface MHC. A recent phase 1 trial tested the efficacy of IMA203, an autologous T cell product engineered to express a TCR recognizing PRAME (a melanoma associated antigen) in the context of HLA-A*02:01, in patients with advanced cancers with no standard options left [105]. Despite unfavourable characteristics (e.g. high LDH, liver/brain metastases, multiple lines of previous treatment) in this difficult to treat patient population, the study reported impressive confirmed and unconfirmed response rates of 37.5% and 75%, respectively in patients with melanoma (*n* = 16). Importantly, many of these responses were also durable. IMA203 treatment was well tolerated with most grade 3/3+ toxicities attributed to lymphodepleting chemotherapy as opposed the cell therapy product itself [105]. On the back of these encouraging data, a confirmatory phase 3 trial is now actively recruiting (NCT06743126).

The hostile tumour microenvironment (TME) of solid cancers has also been proposed to limit the infiltration, function and survival of adoptively transferred T cells [106, 107]. Rather than employing conventional αβ T cells, we and others have instead proposed utilizing unconventional γδ T cells [108, 109]. The intratumoural presence of γδ T cells has been shown to better predict survival than that of αβ T cells in multiple cancer types [110–115], including melanoma [116]. Moreover, γδ T cells display many key traits which may overcome a hostile TME. For example, these cells are often already resident in pre-malignant tissues [111, 113, 114, 117], display long-term persistence after adoptive transfer [118] and are seemingly resistant to exhaustion [116,119–121]. Importantly, these cells can also recognize tumour cells that have evolved to evade conventional αβ T cells (e.g. low mutational burden, loss of MHC) [108, 116]. Early-phase clinical studies utilizing γδ T cells have demonstrated a good safety profile although it remains too early to tell if these cells are effective [108].

### Intratumoural immunotherapies

The success of TVEC, notably its capacity to induce abscopal remissions with only minimal systemic irAEs, clearly supports the utility of intratumoural delivery. Thus, a second-generation oncolytic virus, vusolimogene oderparepvec (RP1), has been developed as a direct successor to TVEC. Indeed, RP1 is essentially a modified TVEC with the addition of a transgene encoding a highly fusogenic form of the envelope glycoprotein of gibbon ape leukaemia virus which confers significantly increased oncolytic capacity [122]. A phase II trial of intratumoural RP1 in combination with anti-PD-1 in 140 patients with anti-PD-1 resistant melanoma found an impressive 33% objective response rate [123]. Whilst the addition of RP1 was predictably associated with low-grade injection site reactions, the rate of grade 3/3 + treatment-related adverse events was modest (∼13%) and comparable to historical rates associated with anti-PD-1 monotherapy. Moreover, compared with TVEC + anti-PD-1 in a similar patient cohort, albeit from a separate phase II trial [87], RP-1 + anti-PD-1 was associated with a numerically superior response rate (∼2–3% versus 33%).

Beyond oncolytic viruses, other locally delivered immunotherapies are also gaining traction. One such example is darumon which is a combination of two immunocytokines (TNFα and IL2, each fused to an antibody fragment targeting fibronectin) delivered by direct intratumoural injection [124]. A recent phase III trial of neoadjuvant darumon versus upfront surgery for resectable melanoma (PIVOTAL, NCT02938299) showed a significant improvement in relapse-free survival in favour of darumon (hazard ratio = 0.59) [124]. Notably, over a third of patients included in the PIVOTAL study had received previous adjuvant therapy for melanoma with the majority of these having received CPI therapy. Moreover, the authors reported a very low rate of irAEs (2.5%) although the criteria for classifying adverse events as irAEs was not well described. Both darumon and RP1 have demonstrated encouraging efficacy in the anti-PD-1 resistant setting. Combined with potentially lower rates of systemic irAEs, these drugs are viable options for patients with anti-PD-1-resistant disease and/or those with poorly controlled autoimmune conditions, which may be exacerbated by systemic immunotherapies.

### Targeting the microbiome

Unfortunately, a substantial proportion of patients with advanced melanoma do not benefit from CPI therapies [69, 77, 125]. Nonetheless, these therapies can clearly cure a subset of patients and understanding the determinants of this CPI responsiveness and mechanisms of CPI resistance should extend the utility of these tried-and-tested drugs.

One area of intense research has been around the potential impact of the microbiome on CPI responsiveness/resistance and cancer immunosurveillance in general. The microbiome comprises trillions of bacteria, archaea, fungi, protozoa, and viruses that constitutively populate cutaneous and mucosal barrier tissues. This microbial community vastly outnumbers the cells of its mammalian host and encodes >10 fold more unique genes [126]. The influence of the microbiome on its host is exerted locally via interactions with barrier tissues and systemically via microbial metabolites that are absorbed into the host circulation and delivered to distant organs [127]. Whilst the microbiome is implicated in a wide array of critical host functions, its role in regulating systemic immune development and function is of particular interest with respect to modulating anti-cancer immune responses during CPI therapy [128]. In support of such a role are several studies showing that mice raised in germ-free (GF) facilities or exposed to broad-spectrum antibiotics display striking attenuation of CPI-responsiveness that is rescued by oral microbial gavage [129, 130]. In humans, several studies have found that CPI-responsiveness segregates with microbiome composition [131–133]. Moreover, exposure to antibiotics at the start of CPI therapy has also been associated with reduced benefit in patients with melanoma and other cancers [134]. Notably, transplantation of faecal microbiota from CPI-responsive versus CPI-refractory human donors into avatar GF mice appears to transfer the donor’s CPI-response phenotype into the recipient mice [131, 132]. Thus, the composition of human gut microbiota is thought to be both a critical and modifiable determinant of CPI-responsiveness.

Several early phase clinical trials have demonstrated that faecal microbiota transplantation (FMT) in the setting of advanced or metastatic melanoma is technically feasible and well-tolerated alongside CPI therapy [135–137]. Notably, two studies found FMT from CPI-responsive donors was associated with rescue of CPI-responsiveness in 3/10 patients [135] and 3/15 patients [136] with refractory disease. The use of FMT to augment the efficacy of first-line CPI therapy has also been explored in an early phase trial in which 20 patients with advanced melanoma were treated with encapsulated faecal-microbiota derived from healthy donors in addition to anti-PD-1 therapy [137]. Objective responses were reported in 13/20 patients, with 4 complete responses and 9 partial responses. Although this response rate exceeds the expected response rate for anti-PD-1 therapy alone in this patient population (∼40%), larger randomized and controlled studies are needed to draw firm conclusions. Given the pervasive influence of the microbiome on systemic immune responses, FMT has also been trialled in modulating CPI-induced irAEs where it has demonstrated efficacy in treating refractory CPI-induced colitis [138]. Thus, FMT may eventually be deployed not only to augment/rescue ICI-responsiveness but also to optimize the management of irAEs.

## Concluding remarks

**Figure 3:**
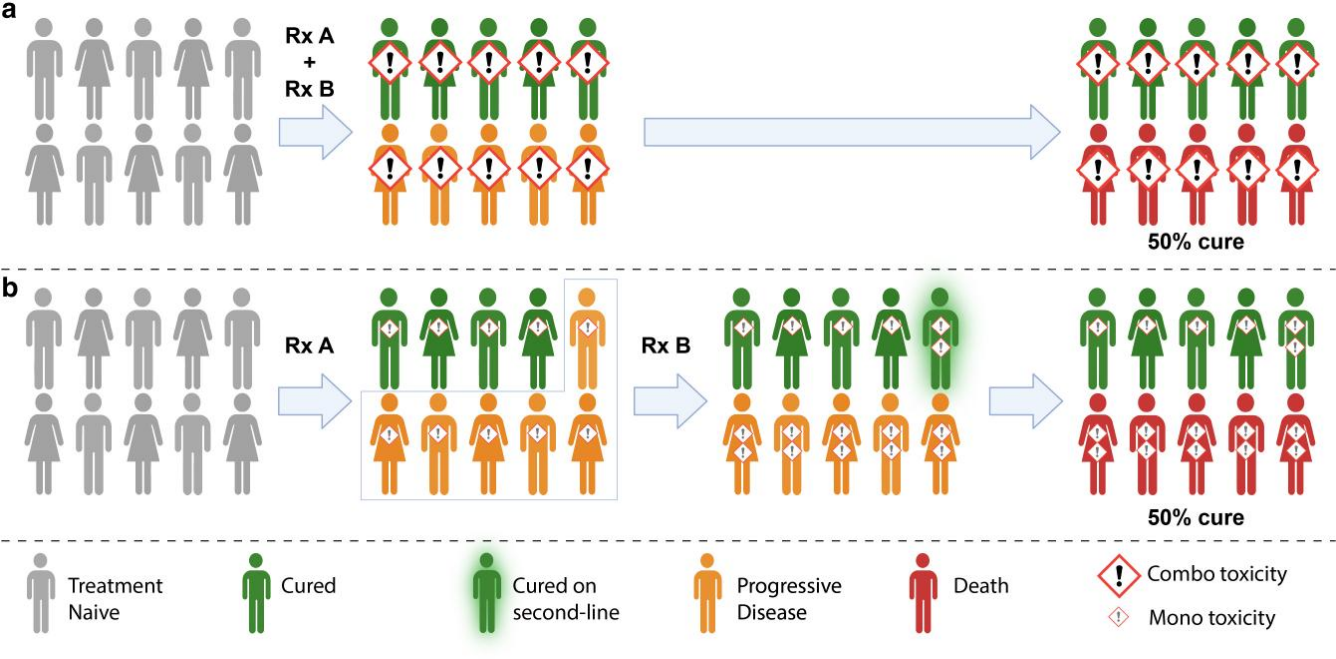
Combination versus sequential immunotherapies. Combination immunotherapies have yet to convincingly demonstrate synergistic efficacy. Current evidence suggests (at best) additive efficacy. **a**) In this scenario, first-line combination therapy with treatment A (Rx A) and treatment B (Rx B) results in an impressive ∼50% of patients being cured. However, all patients would be exposed to substantial combination therapy toxicity, including 5/10 patients who would have had futile treatment and 4/10 patients who would have done just as well with first-line Rx A monotherapy. **b)** On the other hand, first-line Rx monotherapy cures ∼40% of patients. Critically, patients only get exposed to monotherapy toxicity which is considerably less than combination toxicity. Of the 6/10 patients with progressive cancer, some can be cured in the second-line setting with Rx B monotherapy, yielding an overall cure rate of ∼50%. Importantly, the patients who are cured already by first-line immunotherapy are spared compound toxicity whilst the other ∼60% of patients with progressive cancer only experience second-line Rx B monotherapy toxicity. Created in BioRender. Wu, Y. (2025) https://BioRender.com/mosz45t.

Modern immunotherapies, particularly CPIs, have transformed the clinical management of advanced melanoma. These standard-of-care treatments for melanoma are now also being adopted in the management of other cancer types. With the addition of targeted therapies directed against activating BRAF mutations (found in ∼40% of cutaneous melanomas), over half of patients today with advanced cutaneous melanoma will survive for more than 5 years after their initial diagnosis. Moreover, most such patients are likely to be cured of their cancer. In our opinion, this curative potential of immunotherapies and the paucity of evidence for synergistic efficacy [66–68] are important to bear in mind when considering how to combine or sequence these treatments. If combination therapies are at best additive in efficacy, then sequential monotherapies rather than upfront combination may be a better option. A sequential approach would spare many patients the unnecessary toxicity of combination therapy whilst still curing patients who may benefit from addition of a second drug (Fig. 3). Indeed, sequential treatment is a well-established paradigm in the curative management of lymphomas [139]. Nonetheless, many oncologists remain sceptical with a common reservation being that some patients can progress rapidly after first-line therapy and never make it to starting second-line treatment. Whether these patients would have benefitted from first-line combination immunotherapy or whether they simply had aggressive disease and would have progressed regardless of upfront combination is not clear. The lack of overall survival benefit of combination CPIs versus anti-PD-1 monotherapy in all-comer patients [3, 69, 77] suggests the latter, although careful consideration of subgroups (e.g. BRAF mutant melanomas, PD-L1 < 1%) [3, 76] may enrich for some patients more likely to benefit from first-line combination. Unfortunately, CPIs are not independent of each other in their mechanism of action and resistance to one CPI (e.g. via defects in antigen presentation) often confers resistance to other CPIs [24]. Thus, the addition of more CPIs to anti-PD-1, whether in first-line combination or in sequence, is likely to yield only diminishing returns in efficacy. In our opinion, the greatest gains will be from novel immunotherapies with more independent mechanisms of action to CPIs (e.g. TILs, oncolytic viruses). Notwithstanding these caveats, we remain optimistic and as melanoma continues to lead the way in trials of novel immunotherapies, we hope many of the investigational therapies reviewed above will have enabled further survival gains in 5 years’ time.

## Data Availability

No new data were generated or analysed in support of this review.

## Permission to reproduce

Figure 1, Created in BioRender. Wu, Y. (2025) https://BioRender.com/bbudcuu

Figure 2, Created in BioRender. Wu, Y. (2025) https://BioRender.com/3upc37h

Figure 3, Created in BioRender. Wu, Y. (2025) https://BioRender.com/mosz45t
